# Synthesis and Stabilization of Support-Free Mesoporous Gold Nanoparticles

**DOI:** 10.3390/nano10061107

**Published:** 2020-06-03

**Authors:** Laura Juhász, Krisztián Moldován, Petra Herman, Zoltán Erdélyi, István Fábián, József Kalmár, Csaba Cserháti

**Affiliations:** 1 Department of Solid State Physics, University of Debrecen, Egyetem sqr. 1, H-4032 Debrecen, Hungary; juhasz.laura@science.unideb.hu (L.J.); zoltan.erdelyi@science.unideb.hu (Z.E.); 2 Doctoral School of Physics, University of Debrecen, Egyetem sqr. 1, H-4032 Debrecen, Hungary; 3 Department of Inorganic and Analytical Chemistry, University of Debrecen, Egyetem sqr. 1, H-4032 Debrecen, Hungary; moldovan.krisztian@science.unideb.hu (K.M.); herman.petra@science.unideb.hu (P.H.); ifabian@science.unideb.hu (I.F.); 4 Doctoral School of Chemistry, University of Debrecen, Egyetem sqr. 1, H-4032 Debrecen, Hungary

**Keywords:** porous gold nanoparticles, dewetting-dealloying, localized surface plasmon resonance, support-free porous noble metal

## Abstract

Porous gold nanoparticles (PGNs) are usually prepared in an immobilized form on a solid substrate, which is not practical in many applications. In this work, a simple method is reported for the preparation and stabilization of mesoporous gold particles of a few hundred nanometers in size in aqueous suspension. Nanoparticles of Ag-Au alloy were fabricated on CaF${}_{2}$ and Si/SiO${}_{2}$ substrates by the solid-state dewetting method. Silver was selectively dissolved (dealloyed), and the resulting porous gold nanoparticles were chemically removed from the substrate either in a concerted step with dealloying, or in a subsequent step. Nitric acid was used for the one-step dealloying and detachment of the particles from CaF${}_{2}$ substrate. The consecutive use of HNO${}_{3}$ and HF resulted in the dealloying and the subsequent detachment of the particles from Si/SiO${}_{2}$ substrate. The PGNs were recovered from the aqueous suspensions by centrifugation. The Au content of the suspensions was monitored by using elemental analysis (ICP-OES), and recovery was optimized. The morphology and the optical characteristics of the support-free PGNs were analyzed by scanning electron microscopy (SEM), dynamic light scattering spectroscopy (DLS), and near-infrared spectrophotometry (NIR). The obtained PGNs are spherical disk-shaped with a mean particle size of 765 ± 149 nm. The suspended, support-free PGNs display an ideally narrow dipole plasmon peak at around 1450 nm in the NIR spectral region. Thus, the new colloidal PGNs are ideal candidates for biomedical applications, for instance photothermal therapy.

## 1. Introduction

Porous noble metal nanoparticles and thin layers are highly attractive for applications in catalysis, sensing, plasmonics, biochemistry, medical diagnosis, and therapy owing to their unique chemical and optical characteristics. [1,2,3,4,5,6,7,8,9,10]. Not only the high surface/volume ratios but also the advantageous optical properties of the porous gold nanoparticles (PGNs) are attractive and interesting. The plasmonic properties of PGNs are fundamentally different from those of their solid counterparts of the same diameter. Even a well-defined porous particle can yield multiple, infrared-shifted plasmon peaks due to the fragmentation of the bulk metal phase. [11,12,13]. It is also well-known that these 3D structures can be passivated by a few nanometer thick metal-oxide layers, which preserves their morphologies as well as their plasmonic properties after high-temperature heat-treatment [14,15]. Furthermore, it is also an additional tool for tuning the optical properties of PGNs and shifting the wavelengths of the dipole plasmon peaks [14,15,16,17]. However, the presence of a substrate naturally limits the utilization of porous nanoparticles.

Support-free porous noble metal nanoparticles can conveniently be administered and tracked in biological systems. These properties together with their porosities and optical characteristics form the basis of their biomedical applications, such as drug delivery [18,19], drug-screening [20], drug encapsulation [21,22], gene delivery [23,24,25], photothermal therapy [6,7,8,9,10], diagnostic imaging [26] and ophthalmological applications [27]. Furthermore, the biocompatibility of gold nanoparticles has also been confirmed by numerous cytotoxicity studies [28,29].

Only a handful of methods are reported in the literature for the synthesis of support-free porous gold nanoparticles. Hu et al. prepared colloidal porous gold nanoparticles with hierarchical structures by using the ambient wet-chemical method and colloidal PbS nano-octahedrons [30]. Pedireddy et al. reported the fabrication of zero-dimensional hollow porous gold nanoparticles by using a novel one-step solution phase method at room temperature [31]. Park et al. prepared porous gold nanoparticles by using nanoimprint lithography, and investigated their photothermal and photoacoustic properties [32]. Liu et al. have fabricated support-free porous nanoparticles, but Au-Ag alloys and not pure Au, in a thin SiO${}_{2}$ cell by using the dewetting-dealloying method [33].

The ultimate goal of our work was to develop a method for detaching specific dewetting-dealloying fabricated mesoporous gold nanoparticles from the substrate and stabilizing the particles in aqueous suspension. With this approach, we aim to broaden the field of the applications of PGNs and offer a new method for their preparation.

## 2. Materials and Methods

### 2.1. Sample Preparation

Porous gold nanoparticles were synthesized on Si(111)/SiO${}_{2}$ and also on 1 cm × 1 cm CaF${}_{2}$ (111) substrates (CRYSTAL GmbH, Berlin, Germany ) as follows. First, 6 nm gold and subsequently 16 nm silver layers were deposited on the substrates by using the magnetron sputtering method [16,17,34]. The sputtering rates of gold and silver were 0.425 nm/s and 0.65 nm/s, respectively. Depositions were carried out at 5 × $10^{-3}$ mbar of Ar. Gold-silver alloy nanoparticles were fabricated from these films by the solid state dewetting method. The thin films were annealed at 850 °C in dynamic atmosphere (95% Ar + 5% H${}_{2}$) with 0.3 L/min flow rate for 30 min. The resulting gold-silver nanoparticles are spherical and ca. 350 nm in diameter. The ratio of Ag and Au in the alloy particles is set by the sputtering parameters.

Two methods are presented in this work for preparing colloidal PGNs in aqueous medium: a one-step process was developed for detaching PGNs synthesized on CaF${}_{2}$ substrate and a two-step process for detaching PGNs synthesized on Si/SiO${}_{2}$ substrate. It is well-known that silver dissolves in concentrated nitric acid (65 wt.% HNO${}_{3}$ (VWR, Debrecen, Hungary)), but gold remains intact. Thus, by treating Ag-Au alloy nanoparticles with cc. HNO${}_{3}$, porous gold particles form due to dealloying on the surface of the substrate with ca. 10–50 nm pore sizes depending on the alloy composition and the dealloying conditions [15,34]. In order to achieve the concerted formation and detachment of PGNs, Au-Ag nanoparticles prepared on the 1 cm × 1 cm CaF${}_{2}$ substrate were immersed into 1.5 mL 65 wt.% HNO${}_{3}$ for 30 min. This treatment selectively dissolves silver from the alloy nanoparticles and simultaneously removes PGNs from the CaF${}_{2}$ substrate due to the etching of the surface. Accordingly, this is a one-step process for the preparation of PGNs.

When Ag-Au nanoparticles are prepared on Si/SiO${}_{2}$ substrate, the treatment with cc. HNO${}_{3}$ does not detach the porous gold particles. After dissolving silver from the alloy nanoparticles (by the treatment with 1.5 mL 65 wt.% nitric acid for 30 min), the samples were immersed into 1.5 mL 10 wt.% HF (Spectrum-3D, Debrecen, Hungary) solution. Hydrogen fluoride etches the surface of the Si/SiO${}_{2}$ substrate and removes the PGNs. These two steps constitute the two-step process for the preparation of PGNs. During the chemical treatments, the samples were sonicated in a supersonic bath at room temperature. Reference samples of PGNs were synthesized on sapphire substrate by using the same dewetting method and dealloying by cc. HNO${}_{3}$.

**Scheme 1:**
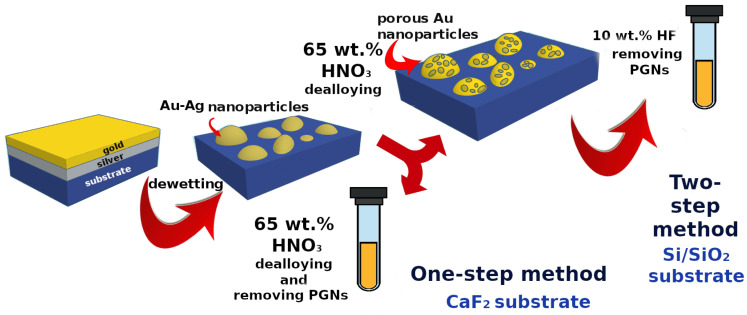
Schematic representation of the different preparation methods of colloidal PGNs.

The detaching processes provide porous gold nanoparticles suspended either in nitric acid or in hydrogen fluoride solution. Separation and purification of PGNs from the acid were carried out by using centrifugation, which was performed with an Orto Alresa Digicen 21 R instrument (Madrid, Spain). The centrifugation conditions were optimized in order to maximize the yield and minimize possible morphological changes in PGNs. It was found that a significant portion of the PGNs was pressed into the bottom of the centrifuge tube in the case of plastic (PP) centrifuge tubes, and this significantly hindered recovery. In order to avoid the loss, glass centrifuge tubes were used. The glass centrifuge tubes were soaked in chromosulfuric acid for 2 h, thoroughly washed with deionized water, and dried before using them for the centrifugation of PGNs. In the case of the HF containing PGN suspensions, plastic centrifuge tubes were used to avoid the mineralization of the glass tubes. The optimal protocol of centrifugation was established, as follows. The original suspension was centrifuged for 15 min at 1272× *g* then 1.3 mL supernatant (87% of the original volume) was removed and the residue was diluted to 2.0 mL with deionized water. The new suspension was sonicated for 10 min in order to resuspend the PGNs. This process yields a 10-fold dilution of the original reactants (acids) with maximum recovery of PGNs. When necessary, the centrifugation process could be repeated in order to change the electrolyte or concentrate the suspension with minimal loss of PGNs, as described in the Results and Discussions section. The schematic representation of the steps of the different preparation methods is given in Scheme 1.

### 2.2. Sample Characterization

The morphology of the PGNs was studied by scanning electron microscopy (SEM) with HITACHI-S4300-CFE (Hitachi High-Technologies Corp., Csijoda-ku, Tokyo, Japan) and Thermo Fisher Scientific-Scios 2 instruments (Waltham, MA, USA). Low accelerating voltage (5 kV & 2 kV) and small working distance (≤7 mm) were used in order to achieve high resolution and good surface sensitivity. The nanoparticles were investigated on the surfaces of substrates after the dewetting-dealloying process and after detachment and recovery. 200 μL of aqueous PGN suspension was desiccated on Si or sapphire substrate for additional measurements. National Instruments Vision software was utilized for calculating the size distribution of the nanoparticles. The secondary electron (SE) images were evaluated.

Dynamic light scattering (DLS) measurements were performed with a Malvern Zetasizer Nano ZS instrument (Worcestershire, UK) equipped with a 632.8 nm He-Ne laser and operating at an angle of 173°. 1.5 mL of each suspension was measured in single-use polystyrene cuvettes with a pathlength of 10 mm. The measurements were made at a position of 4.65 mm from the cuvette wall with an automatic attenuator and at a controlled temperature of 25 °C. For each sample, 10–15 runs of 10 s were performed, with 3 repetitions. Measurement data were collected and evaluated using the Zetasizer software. The Z-average diameter (Z-ave) and the particle size distribution curves were calculated directly from the autocorrelation function by using the built-in CONTIN algorithm [35,36].

The Au content of the PGN suspensions was measured by elemental analysis using inductively coupled plasma optical emission spectrometry (Agilent Technologies ICP-OES 5100, Australia). The initial acidic suspensions, and after each centrifugation step the supernatants and resuspended samples were analyzed by ICP-OES in order to quantify the initial and the recovered amount of Au. Three different sample preparation methods were used and compared to the elemental analysis of Au. In the first method, samples were directly nebulized (without digestion) into the atomizing unit of the ICP-OES instrument. In the other two methods, atmospheric and microwave-assisted (Milestone, Ethos Up, Sorisole, Italy) wet digestions were applied to prepare the samples for ICP-OES analysis. In the first digestion process, 200 μL of the suspension was measured into an acid-pretreated (cc. HNO${}_{3}$) beaker and digested on an electric hot plate with 2.0 mL of aqua regia at 80 °C for 4 h. After digestion, the sample was diluted with Milli-Q water to a final volume of 3.0 mL. In the microwave-assisted sample preparation method 200 μL of the suspension was measured into a sealable PTFE vessel and digested with 0.8 mL aqua regia. The samples were microwave heated to 200 °C and digested for 20 min. The vessels were allowed to cool down and the samples were brought to a 3.0 mL final volume with Milli-Q (Merck, Darmstadt, Germany) water.

The Au concentrations of non-digested and digested samples (initial and centrifuged solutions, supernatants) were measured by ICP-OES by using the same protocol. Four-point calibration was applied (0–200 μg·L${}^{-1}$), diluted from a monoelement Au standard solution of 1000 mg·L${}^{-1}$ (Scharlab, Debrecen, Hungary). Emission intensity values were collected at 3 different wavelengths of Au. The concentrations were calculated based on the optical lines that gave the best signal-to-background ratio. The concentrations of 3 independent parallel samples were averaged, and the relative standard deviation (RSD) was calculated. The ICP-OES experimental parameters are given in Table 1.

The most representative results were obtained by using the microwave-assisted sample preparation method. With the utilization of microwave-assisted digestion, 102 ± 3% of the theoretically calculated amount of Au was quantified in the acidic PGN suspensions. Therefore, all further analytical measurements were performed by using this protocol. Applying atmospheric wet digestion or directly nebulizing the PGN suspensions yielded ca. 50% of the Au concentrations measured by using the microwave-assisted digestion method for identical samples.

The optical absorption spectra of PGNs on substrate, as well as the spectra of PGN suspensions were measured by a SHIMADZU 3600 UV-Vis-NIR ( SHIMADZU Corp., Kyoto, Japan) spectrophotometer in the spectral range of 350–1750 nm. The suspensions were loaded into a high precision cell with 1.00 mm optical pathlength and 350 μL volume. Measurements were performed in each step of the synthesis and purification process. The baselines for the PGN suspensions were recorded by using the corresponding electrolyte solutions (HNO${}_{3}$ or HF solutions), or water.

## 3. Results and Discussion

Because the use of glass centrifuge tubes greatly elevated recovery, representative recovery values are given for the one-step detaching of PGNs starting from CaF${}_{2}$ supported particles by using cc. HNO${}_{3}$. The initial PGN suspensions produced by the acidic treatment of supported nanoparticles contained 7.37 ± 0.84 μg Au. The amount of Au initially deposited on the surface of the substrate is estimated to be 7.2 μg by taking into account the sputtering parameters and estimating the density of the sputtered thin film to be 80% of that of bulk Au. Accordingly, the amount of material in the particles detached from the CaF${}_{2}$ support perfectly matches the amount of material deposited by sputtering, which shows the excellent efficiency of the detachment method. (SEM images of spent supports recorded after the detachment confirm the complete removal of the nanoparticles.) The Au contents of the suspensions were determined after each centrifugation step and compared to that of the initial acidic suspension. 6.26 ± 0.72 μg Au was recovered after the first centrifugation step, and the second round of centrifugation decreased the yield to 5.11 ± 0.58 μg Au. These values correspond to 88% and 69% of the amount of Au originally detached from the substrate, respectively.

**Figure 1:**
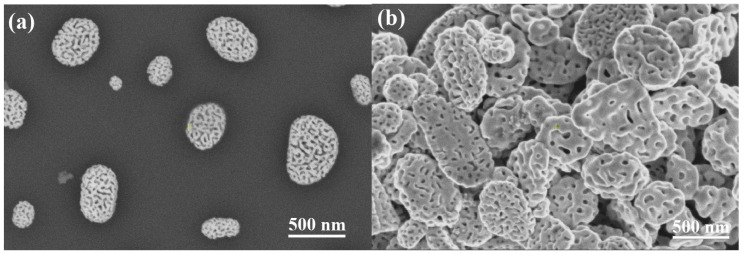
(**a**) SEM images of porous gold nanoparticles (PGNs) prepared by the dewetting-dealloying method on Si/SiO${}_{2}$ substrate. (**b**) SEM images of support-free PGNs chemically detached from the substrate. The suspension of the support-free particles was desiccated on Si for imaging. It is notable that the support-free PGNs are spherical disk shaped.

Figure 1 shows representative SEM images of supported PGNs and support-free PGNs chemically detached from the substrate.

**Figure 2:**
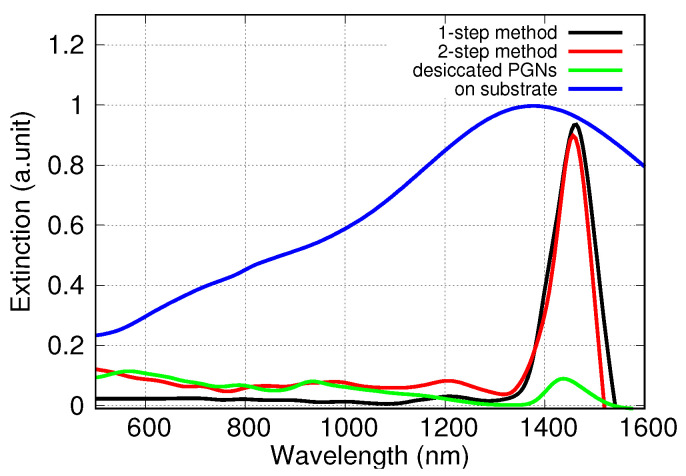
Optical absorption spectra of supported PGNs on sapphire substrate and support-free PGNs suspended in water or desiccated in sapphire. (Black and red lines: Spectra of the support-free PGNs in water prepared by the one-step and two-step detachment methods, respectively. Green line: Spectrum of support-free PGNs desiccated on sapphire. Blue line: Reference spectrum of supported PGNs on sapphire.).

**Figure 3:**
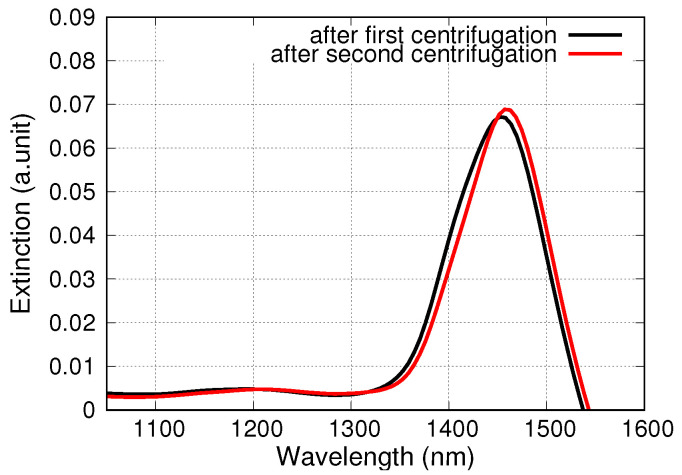
Optical absorption spectra of support-free PGNs prepared by using the one-step detachment method starting from particles supported on CaF${}_{2}$. Spectra were recorded after the first and second centrifugation steps. The two spectra overlap.

Figure 2 shows the optical absorption spectra of support-free PGNs in water prepared by the one-step and the two-step detachment methods and recovered by centrifugation. The overlap of these spectra is excellent. It is clearly seen that the dipole plasmon peak is located at ca. 1450 nm. It is notable that the position and the shape of the major absorption peak do not change after the repeated centrifugations of the PGN suspensions, as seen in Figure 3.

In order to compare our results with previous findings, PGNs were prepared on sapphire substrate as reference material by using the common dewetting-dealloying procedure. The optical absorption spectra of PGNs on sapphire substrate were measured by using the same conditions as in the case of the suspensions of the support-free PGNs (Figure 2). The dipole peaks of the supported and the support-free particles are situated at the same wavelength range, but the widths of the peaks are significantly different. It is clearly seen in Figure 2 that the dipole plasmon peak of the supported PGNs is much wider than the peak of the support-free PGNs. This indicates that the size distribution and/or the morphology of the PGNs change in an advantageous manner during the detachment and recovery processes [37,38].

**Figure 4:**
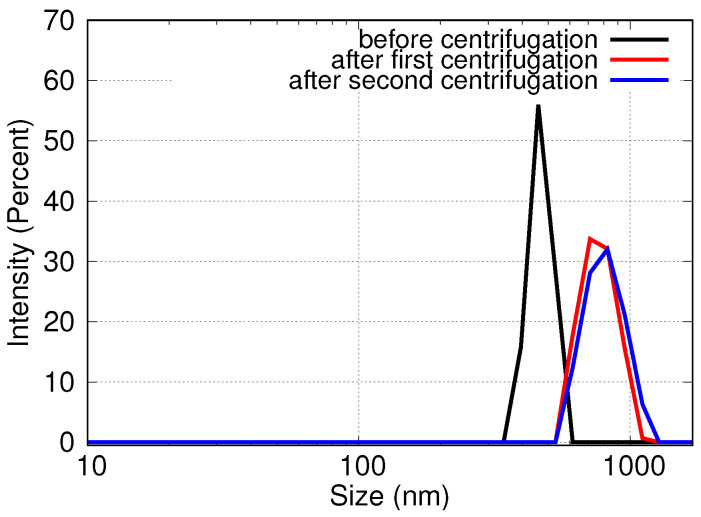
Particle size distribution curves of support-free PGNs measured in their suspensions by DLS after each recovery step. The size distribution of PGNs is altered in the first centrifugation step, but the second centrifugation does not affect further the size of the PGNs.

SEM and DLS measurements confirm this modification in terms of the size and shape of the support-free PGNs. The average diameter of the supported particles was calculated by analyzing SEM (SE) images using the NI Vision software. The average diameter of gold-silver alloy nanoparticles is 316 ± 136 nm on CaF${}_{2}$ substrate and 370 ± 100 nm on Si/SiO${}_{2}$ substrate. The mean particle size of the detached PGNs is significantly larger and their size distribution is significantly narrower than that of the supported particles according to the DLS analysis of the PGN suspensions (Table 2 and Figure 4) and the SEM analysis of the desiccated suspensions (Figure 1).

Specifically, the mean size of the detached PGNs measured by DLS before centrifugation is somewhat larger than the particle size calculated by the image analysis of the SEM pictures of the same particles still on the surface of the substrate (first 2 rows of Table 2). This difference is only apparent, since the DLS measurement is sensitive to the hydrodynamic radius of the suspended particles, and this is naturally larger than the physical size of the non-hydrated particles. On the other hand, the DLS measurements show that the mean size of the suspended particles is larger after centrifugation than in the original acidic suspension after detachment. This change in the size of the PGNs is advantageous because it causes the significant narrowing of the dipole plasmon optical absorption peak (cf. Figure 2). Size fractionation is not a feasible explanation for the increase of the mean particle size of PGNs during centrifugation, because the elemental analysis of the suspensions showed that the loss of material is very low in the centrifugation steps.

The most feasible explanation for the increase of the mean particle size is the aggregation of the particles and the minor alteration of the shape of the particles due to centrifugation. Aggregation is natural when such nanoparticles are centrifuged that are not stabilized by surfactants or strong electrolytes. Furthermore, the SEM images of desiccated suspensions show somewhat clustered and flattened particles (cf. Figure 1b). It is important to emphasize that, in spite of these observations, multiple rounds of centrifugation steps do not cause a gradual change either in the size or in the morphology of the support-free PGNs (cf. Table 2 and Figure 3 and Figure 4). The PGNs in the suspensions are ideally stable after the first centrifugation process, and the PGNs can be processed further without any change in particle size distribution or particle morphology. The stability of the suspension is confirmed by optical spectroscopy measurements, as well, since the NIR spectrum of the suspended particles is the same after 1 and 2 rounds of centrifugation steps, as shown in Figure 3. Thus, the advantageous optical properties of the colloidal PGNs are stabilized by the applied recovery process.

An additional experiment was performed to further study the optical properties of the support-free PGNs. An aliquot of a concentrated PGN suspension was desiccated on a sapphire substrate and the optical absorption spectrum of the dry particles was measured. As Figure 2 shows, the spectrum of the dry particles displays the same narrow dipole plasmon peak as the original suspension. This highlights that neither the hydration level of particles nor the presence of non-conducting support changes the optical properties of PGNs. This result is consistent with our previous findings, i.e., the shape and the size distribution of the PGNs are the key characteristics that determine the fundamental optical properties of the support-free particles.

## 4. Conclusions

A simple method is presented for the synthesis and stabilization of porous gold nanoparticles (PGNs) in water. Two processes were developed and optimized for the production of support-free PGNs, one starting from particles initially supported by CaF${}_{2}$, and another starting from particles initially supported by Si/SiO${}_{2}$. Porous gold particles were fabricated on CaF${}_{2}$ and Si/SiO${}_{2}$ substrates using the dewetting-dealloying method, and afterward removed chemically by using nitric acid and hydrogen fluoride, respectively. The representative recovery of 88% was achieved starting from CaF${}_{2}$ supported particles. When comparing the 2 processes, it is evident that the simplicity, the absence of HF, and the greater recovery make the process starting from CaF${}_{2}$ supported particles superior. The mean diameter of the PGNs recovered by centrifugation is somewhat larger and the size distribution of the particles is narrower compared to the parent particles on the substrate. Centrifugation also causes minor alteration of the shape, i.e., the flattening of the spherical particles. Owing to the changes in size and shape, the dipole plasmon peak of the support-free PGNs (at ca. 1450 nm) is significantly narrower compared to that of the supported particles. It is also confirmed that dehydration does not cause any alteration in the optical spectrum of the support-free particles. These findings provide potential methods for synthesizing and stabilizing porous gold nanoparticles suspended in water with narrow dipole plasmon peaks, which might broaden the field of their applications [39]. The intensive optical absorption of the new PGNs at around 1450 nm combined with the biocompatible nature of these types of nanomaterials makes them ideal for both biomedical imaging, and in the case of the advantageous organ-specific accumulation, targeted photothermal therapy by using NIR light sources. Colloidal PGNs can be utilized as advanced drug delivery systems. A recent example is the realization of the illumination triggered the release of the active ingredient deposited in PGNs [40]. Another advanced application of colloidal PGNs is sensor technology. Porous cubical Au nanoparticles were successfully utilized as receptor materials for membrane-type surface stress sensors [41].

**Figure 5:**
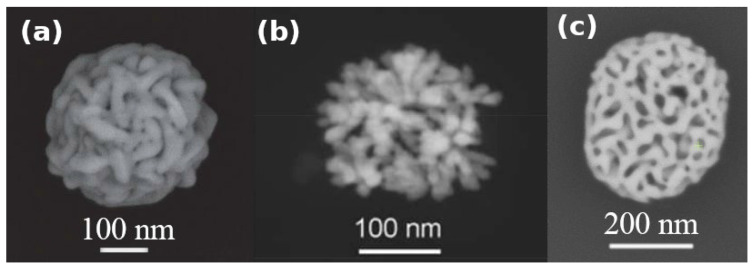
SEM images of colloidal PGNs prepared by using fundamentally different methods: (**a**) Colloidal PGNs prepared by Pedireddy et al. [31] (**b**) Colloidal PGNs prepared by Hu et al. [30] (**c**) Colloidal PGNs prepared by using the dewetting-dealloying method reported in this paper. Figures were reproduced by permission of Springer Nature and RSC Publishing.

It is important to emphasize that the morphologies and the structures of the PGNs prepared previously by Pedireddy et al., and Hu et al. are significantly different from the support-free PGNs reported in this works, as seen in Figure 5. Accordingly, these different synthetic methods are not just different ways to produce similar colloidal particles, but they lay the foundations for designing and altering the fundamental structures of PGNs to ensure the best performance in the targeted application. The structures of these 3 types of porous gold nanoparticles are fundamentally different due to the fundamentally different preparation strategies. The primary building blocks of the particles synthesized by Pedireddy et al. are threads, and the pores are defined by these entangled threads. The PGNs synthesized by Hu et al. are dendritic in structure. The nanoparticles reported in this paper are built from a continuous backbone that are fragmented by spherical pores with practically no focal points or concave structural regions. Thus, the fine-tuning of these different structural elements are expected to change the properties of the PGNs by fundamentally different mechanisms.

## Figures and Tables

**Table 1:** Measurement parameters of the ICP-OES method for Au determination.

replications	3
pump speed	12 rpm
uptake delay	15 s
read time	5 s
RF power	1.20 kW
stabilization time	15 s
viewing mode	axial
nebulizer flow	0.70 L/min
plasma flow	12.0 L/min
aux flow	1.00 L/min
make up flow	0.00 L/min
viewing height	8 mm

**Table 2:** Mean particle size of porous gold nanoparticles (PGNs) at the different stages of synthesis. The size distribution of the initial supported particles was calculated by the analysis of their SEM (SE) images. The suspensions of support-free PGNs detached from CaF${}_{2}$ substrate were analyzed by DLS. Standard deviations are calculated from triplicate measurements. Representative particle size distribution curves measured in the suspensions of PGNs by DLS are given in Figure 4.

Stage of Synthesis	Diameter (nm)
Diameter of supported PGNs	316 ± 136 (image analysis)
Hydrodynamic diameter of support-free PGNs in the original suspension	447 ± 64 (DLS)
Hydrodynamic diameter of support-free PGNs after 1 round of centrifugation	765 ± 149 (DLS)
Hydrodynamic diameter of support-free PGNs after 2 rounds of centrifugations	851 ± 110 (DLS)
